# Decoding the regulatory networks of *Proteus mirabilis* under succinic acid stress: a multi-omics approach

**DOI:** 10.3389/fcimb.2025.1650340

**Published:** 2025-10-03

**Authors:** Aoyu Yang, Yuqing Cai, Ziyi Zhang, Yafang Xu, Chen Shen, Wei Wang, Haiqing You, Shanshan Sha, Huajun Li, Xiancheng Li

**Affiliations:** ^1^ Department of Urology, The Second Affiliated Hospital of Dalian Medical University, Dalian, China; ^2^ Department of Microecology, College of Basic Medical Sciences, Dalian Medical University, Dalian, China; ^3^ Department of Microbiology, Department of Biochemistry and Molecular Biology, College of Basic Medical Sciences, Dalian Medical University, Dalian, China

**Keywords:** multi-omics, succinic acid, biofilm formation, proteus mirabilis, urinary tract infection

## Abstract

*Proteus mirabilis*, a major catheter-associated urinary tract infection pathogen, forms antibiotic-resistant crystalline biofilms. Our study demonstrates succinic acid’s multimodal inhibition of *P.mirabilis* via multi-omics analyses. At 15 mM, succinic acid reduced bacterial growth (≥70%) and biofilm formation (≥50%). Metabolomics revealed that succinic acid treatment induces dysregulation in the tryptophan and arginine metabolism, nucleotide biosynthesis, and tricarboxylic acid cycle in *P.mirabilis*. Transcriptomics revealed downregulated ribosomal genes, oxidative phosphorylation, and efflux pumps, alongside upregulated arginine transport. Proteomics showed suppression of T6SS virulence factors and iron acquisition proteins. We propose that succinic acid reduces K6 acetylation of the histone-like nucleoid structuring protein, enhancing its oligomerization to repress T6SS genes and inhibit biofilm formation. By targeting metabolism, virulence, and stress adaptation, succinic acid circumvents single-target resistance, offering a strategy to combat multidrug-resistant *P.mirabilis* through biofilm disruption and pathogenicity suppression.

## Introduction

**Figure 1:**
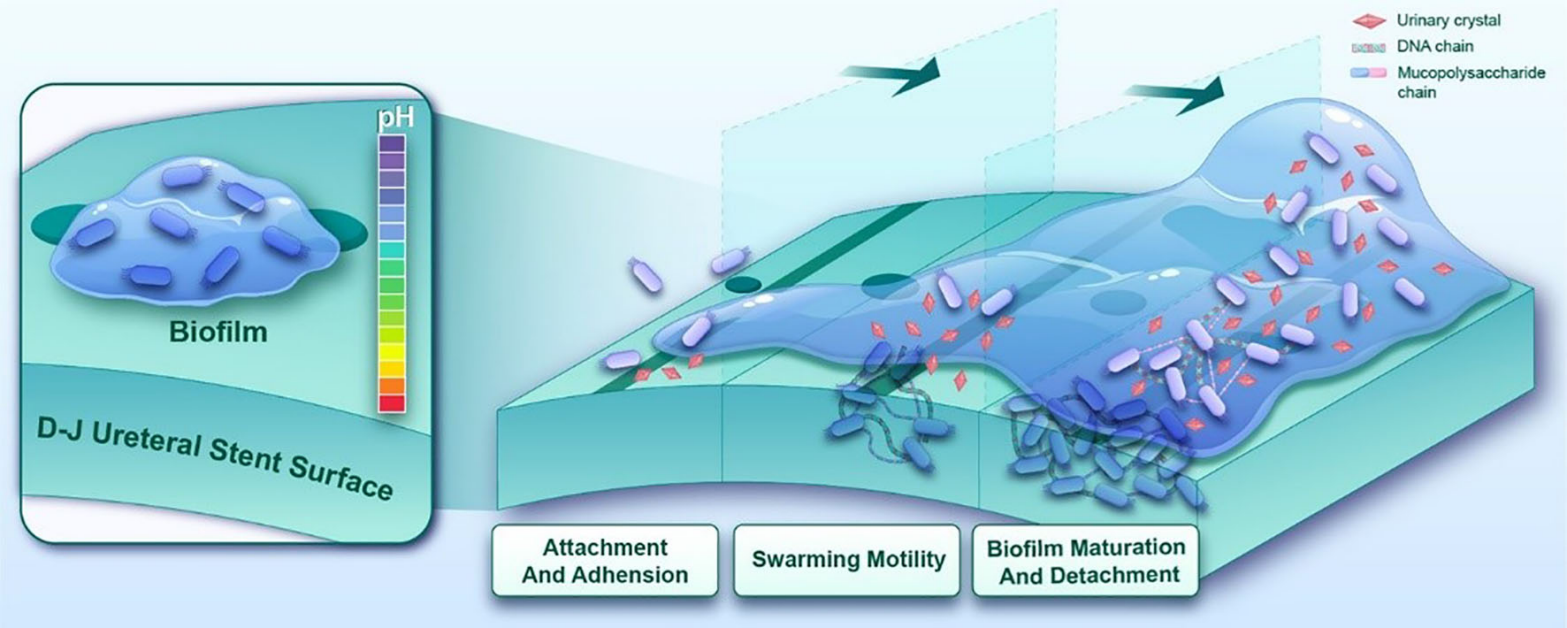
The crystallization biofilm formation process of *P.mirabilis* on ureteral stent surfaces progresses through three distinct phases. Following initial colonization mediated by fimbrial adhesins, quorum sensing-regulated swarming motility is initiated to promote bacterial aggregation and proliferation. Concurrently, urease-mediated urea hydrolysis elevates microenvironmental pH, accelerating mineral crystallization and deposition. These coordinated biological processes ultimately culminate in the development of mature crystal-embedded biofilm.

*Proteus mirabilis*, a Gram-negative bacillus, represents one of the most prevalent pathogens in catheter-associated urinary tract infections (CAUTIs), ranking immediately after *Escherichia coli* and *Klebsiella pneumoniae* in frequency (Sokhn et al., 2020), particularly among elderly patients (Chakkour et al., 2024), and constitutes the predominant causative agent (10%-44%) in double-J stent-associated urinary tract infections (DJUTIs) (Hunt et al., 2024). The pathogenicity of this organism relies on multiple virulence factors, including urease production, adhesins expression, and flagellar motility (Yang et al., 2024). Distinct from other CAUTI pathogens, *P.mirabilis*-induced infections frequently promote urinary calculus formation through urease-mediated urea hydrolysis. This process generates ammonia that elevates urinary pH, facilitating crystal nucleation and growth, thereby exacerbating infection complexity and therapeutic resistance. Furthermore, this organism forms crystalized biofilms through secretion of extracellular polymers (e.g., polysaccharides, proteins), creating mineral-organic composite structures (Awonusi et al., 2022). In clinical practice, indwelling double-J (D-J) stents (retention period: 2 weeks to 1 year) (Wang et al., 2023) provide optimal surfaces for bacterial adhesion and colonization in ureteral obstruction patients. The crystalized biofilms of *P.mirabilis* not only confer resistance to antibiotics and host immune defenses, but also induce stent encrustation and infectious stone formation (Caldara et al., 2025) (**Figure 1**). Biofilm-mediated urinary tract infections demonstrate high recurrence rates and elevate risks of ureteral injury, drainage dysfunction, and renal failure (Prywer et al., 2023). Therefore, inhibiting crystalized biofilm formation constitutes a critical strategy for preventing and managing these infections.

However, the persistent nature of established biofilms poses significant therapeutic challenges. Studies have demonstrated that traditional antibiotics exhibit poor penetrability through *P.mirabilis* biofilms due to extracellular polymeric substances (EPS) entrapment, the biofilm matrix restricts antibiotic diffusion via physical barriers and reduces antimicrobial susceptibility by inducing bacterial metabolic dormancy (Lebeaux et al., 2014). Although current therapies attempt biofilm prevention through early-stage interventions like silver-ion coatings and antibiotic-impregnated catheters (Jacobsen and Shirtliff, 2011), their efficacy remains limited by low silver elution rates and protective crystal layer formation on catheter surfaces. These limitations underscore the critical need for novel biofilm inhibitors targeting *P.mirabilis* in CAUTI management. The development of non-antibiotic antimicrobial agents with specific antibiofilm properties has become an urgent research priority. Compounding this challenge, the escalating antimicrobial resistance in *P.mirabilis* has reached alarming proportions. This pathogen demonstrates heterogeneous resistance profiles across multiple drug classes, significantly complicating clinical treatment strategies. In recent years, an increasing number of studies have documented the emergence of carbapenem-resistant *P.mirabilis (* Bedenić et al., 2025). Carbapenems as a critical last-line treatment option for severe bacterial infections, the occurrence of infections caused by carbapenem-resistant *Enterobacterales* poses a significant clinical challenge, often resulting in limited therapeutic options and high mortality rates (Bassetti et al., 2025). The overuse of antibiotics further exacerbate the development of antimicrobial resistance. Therefore, the identification and development of novel antimicrobial agents with mechanisms of action distinct from those of conventional antibiotics are important for both mitigating the emergence of resistant pathogens and providing effective treatment options against multidrug-resistant infections.

Succinic acid, an organic acid serving as a crucial metabolite in both host organisms and microbial processes, has recently garnered attention for its antimicrobial properties. The antimicrobial mechanism of organic acids involves membrane penetration followed by intracellular dissociation into anions and protons. Proton accumulation induces cytoplasmic acidification, while anion accumulation disrupts metabolic functions, increases osmotic pressure, and ultimately leads to bacterial cell death (Alakomi et al., 2000).

**Figure 2:**
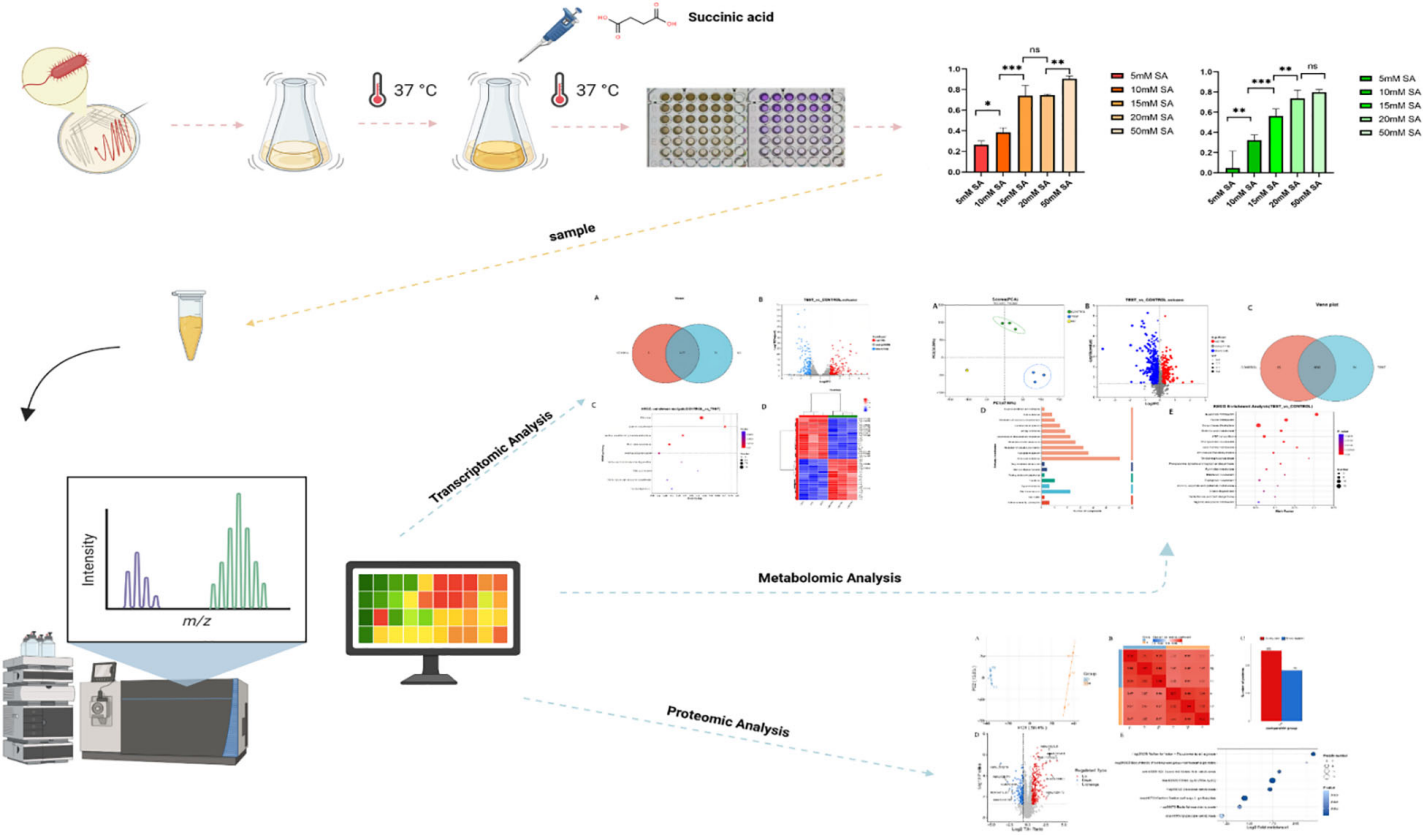
The flowchart of this study. In our study, Proteus mirabilis was treated with different concentrations of succinic acid under 37 °C culture conditions. Subsequently, multi-omics analysis was performed on the treated bacterial samples and the control group to systematically elucidate the molecular mechanism by which succinic acid inhibits P. mirabilis.

Investigations into uropathogens have revealed that organic acids from *Lactobacillus* strains exhibit antimicrobial activity against *P.mirabilis (* Szczerbiec et al., 2023). Notably, succinic acid demonstrates significant growth inhibition and biofilm suppression across all tested *P.mirabilis* clinical isolates. Propidium iodide (PI) staining assays confirmed membrane integrity disruption in *P.mirabilis* following succinic acid treatment. This fluorescent DNA-intercalating agent penetrates compromised membranes, verifying the compound’s membrane-damaging effects (Szczerbiec et al., 2022). Mechanistic studies in *Microcystis aeruginosa* reveal that succinic acid alters membrane permeability through lipid composition modulation. Concurrent metabolic perturbations include: amino acid metabolism inhibition, nucleotide biosynthesis suppression, tricarboxylic acid (TCA) cycle interference, and nitrogen assimilation impairment, ultimately inducing oxidative damage (Chen et al., 2022). These findings suggest a multi-target antimicrobial mechanism involving microbial metabolic network disruption. Despite these evidences of bactericidal and antibiofilm activities against *P.mirabilis*, the precise molecular targets and detailed mechanism of action remain elusive. Therefore, this study employs multi-omics parallel analysis (metabolomics, transcriptomics and proteomics) to systematically elucidate key pathways and regulatory networks underlying succinic acid’s inhibitory effects on *P.mirabilis*, ultimately deciphering its molecular mechanism (**Figure 2**).

## Methods

### Reagents and strains

Succinic acid and all culture reagents, including Tryptic Soy Broth (TSB), agar, Phosphate-Buffered Saline (PBS), methanol, ethanol, and crystal violet, were procured from Dalian Amioke Biological Technology Co., Ltd. Sterile succinic acid solutions (5, 10, 15, 20, 50 mM) were prepared in triple-distilled H2O, sterilized through 0.22 μm membrane filtration, and stored at 4°C. The *P.mirabilis* reference strain ATCC^®^ 29906 was cultured on Tryptic Soy Agar (TSA) plates from -80°C glycerol stock. Single colonies were inoculated into TSB medium and incubated at 37°C with 180 rpm agitation to achieve logarithmic growth phase (OD550 = 0.1, 8×10^8^ CFU/mL) for subsequent experiments.

### Measurement of minimum inhibitory concentration and minimum biofilm inhibitory concentration

The minimum inhibitory concentration was determined using broth microdilution method (Manoharan et al., 2020), defined as the lowest concentration inhibiting ≥70% bacterial growth compared to untreated controls. For the biofilm inhibition assay, succinic acid powder was dissolved in triple-distilled H2O to prepare a filter-sterilized 100 mM stock solution. This stock was then serially diluted to create working solutions at 10, 20, 30, 40, and 100 mM. In the assay, 100 μL of each working solution was added to 100 μL of bacterial suspension in a 96-well plate (n=6), resulting in final succinic acid concentrations of 5, 10, 15, 20, and 50 mM, respectively, after equal volume mixing. Control wells received 100 μL of bacterial suspension mixed with an equal volume (100 μL) of triple-distilled H_2_O. Following 48-hour biofilm formation at 37°C, planktonic cells were removed by triple PBS washing. Biofilms were methanol-fixed (200 μL, 30 min), stained with 0.1% crystal violet (200 μL, 20 min), and destained with absolute ethanol (200 μL). Absorbance at 550 nm was measured spectrophotometrically, with MBIC defined as the concentration achieving ≥50% inhibition calculated by: Inhibition rate (%) = [1 - (OD550 treatment/OD550 control)] × 100%. Independent samples t-test was used to compare differences between every two groups for continuous data. Data analysis was performed using SPSS 25.0, and bar graphs were generated using GraphPad Prism 9.5.1.

### Metabolomics analysis

Single colonies of Proteus mirabilis from Tryptic Soy Agar (TSA) plates were inoculated into Tryptic Soy Broth (TSB) and incubated overnight at 37°C. This process was repeated independently to generate three separate biological cultures. Bacterial suspensions were centrifuged at 6,000×g for 15 min at 4°C, then resuspended in fresh TSB to an optical density at 550 nm (OD_550_) of 0.1 for subsequent experiments. For treatment groups, 7.5 mL of bacterial suspension from each independent culture was mixed with an equal volume of a filter-sterilized succinic acid solution to achieve a final treatment concentration equivalent to the predetermined 15 mM. The mixtures were incubated statically at 37°C for 24 h. Control groups received 7.5 mL of bacterial suspension from each independent culture mixed with an equivalent volume of sterile triple-distilled H_2_O. This resulted in three biologically independent replicates for both the treatment and control groups. Post-incubation, all samples were centrifuged (6,000×g, 15 min, 4°C). The resulting cell pellets were washed once with cold PBS, immediately flash-frozen in liquid nitrogen, and stored at -80°C until metabolomic analysis. The same sample preparation procedure was followed for all subsequent omics analyses. Metabolomic profiling was performed using Thermo Fisher Scientific’s UHPLC-Q Exactive HF-X system. Liquid chromatography separation was achieved with a C18 column (2.1×100 mm, 1.7 μm) maintained at 40°C. Raw LC-MS data were processed through Progenesis QI software (v2.3, Waters) for baseline correction, peak detection, retention time alignment, and generation of an m/z-retention time-intensity matrix. Metabolite identification was achieved by matching MS/MS spectra against HMDB (v5.0) and METLIN (v2023) databases. Data preprocessing involved removing features with >20% missing values across sample groups, imputing remaining missing values with 10% of the minimum observed intensity, and applying total ion current normalization. Quality control filtering excluded variables exhibiting >30% RSD in QC samples, followed by log10 transformation. Multivariate analysis using the ropls package (v1.6.2) in R included PCA for data structure visualization and OPLS-DA with 7-fold cross-validation to assess model robustness. Significantly altered metabolites were selected based on variable importance in projection (VIP) scores >1.0 and Welch’s t-test p value <0.05 after Benjamini-Hochberg correction. KEGG pathway annotation (Release 107.0) and hypergeometric enrichment analysis via scipy.stats (v1.11.1) identified biologically relevant pathways, with statistical significance determined by Fisher’s exact test followed by false discovery rate (FDR) adjustment (q<0.05).

### Transcriptomics analysis

High-quality RNA samples were obtained by extracting RNA from single bacteria and removing genomic DNA using the CTAB method. The rRNA was removed using the RiboCop rRNA Depletion Kit. The mRNA was randomly cleaved into small fragments of 200bp, and reverse transcription was performed using the mRNA as a template to synthesize double-stranded cDNA. When synthesizing the second strand, dUTP was used instead of dTTP. The repaired cDNA was added to the junction, and finally the second strand containing dUTP was removed with UNG enzyme. The library was established using Illumina Stranded mRNA Prep, and double-ended RNA-seq sequencing was performed using NovaSeq™ X Plus. Bioinformatics analysis processes the data generated by the Illumina platform. The gene expression levels were measured using the FPKM and TPM methods. FPKM was calculated based on the number of reads per thousand bases in every million sequences, while TPM was based on the number of reads from a specific transcription per million reads. FPKM and TPM can eliminate the influence of gene length and sequencing depth, thereby enabling the gene expression level to be used for comparison of differences between samples. Differential expression analysis was conducted by obtaining the Read Counts of genes to identify differentially expressed genes and study their functions. The experiment consisted of three biological replicates, and the difference analysis was conducted using DESeq2 software. This analysis was conducted using the software Goatools for enrichment analysis, and the method employed was Fisher’s exact test. To control the calculated false positive rate, four multiple test methods (Bonferroni, Holm, Sidak and false discovery rate) were used to correct the p value. Generally, when the p value was <0.05, it was considered that there was a significant enrichment of this KEGG function.

### Proteomics analysis

Frozen samples were retrieved from -80°C storage and lysed by ultrasonication in ice-cold lysis buffer (8 M urea, 1% protease inhibitor, 3 μM TSA, 50 mM NAM). After centrifugation (12,000 g, 10 min, 4°C), the resulting supernatant was collected for protein quantification using the BCA assay. Equal protein aliquots were precipitated with TCA, washed with acetone, and resuspended in 200 mM TEAB for tryptic digestion (1:50, m/m, overnight). Subsequently, peptides underwent reduction (5 mM DTT, 56°C, 30 min) and alkylation (11 mM IAA, room temperature, 15 min in darkness). Peptide separation was achieved via nanoflow liquid chromatography (NanoElute system) using mobile phase A (0.1% formic acid, 2% acetonitrile in water) and phase B (0.1% formic acid in acetonitrile-water) with a gradient elution (6%-80% B over 20 min, 500 nl/min). Mass spectrometric analysis was conducted on a timsTOF Pro 2 system (ion source voltage: 1.75 kV) in dia-PASEF mode, with MS1 (300–1500 m/z) and MS2 (400–850 m/z) scans at a 7 m/z window width. Protein identification and pathway annotation were performed against the KEGG database using blastp (e-value ≤ 1e-4), retaining the highest-scoring match for each protein.

### Acetylation omics analysis

Frozen specimens were retrieved from -80°C storage and homogenized in lysis buffer (8 M urea, 1% protease inhibitor cocktail, 3 μM TSA, 50 mM NAM) using ultrasonication. After centrifugation, the supernatant was collected for protein quantification. Equal protein aliquots were precipitated with TCA, washed with acetone, and reconstituted in 200 mM TEAB for tryptic digestion (1:50, m/m, overnight at 37°C). Digested peptides underwent sequential treatment with 5 mM DTT (56 °C, 30 min) and 11 mM IAA (room temperature, 15 min in darkness). The peptides were then dissolved in IP buffer (100 mM NaCl, 1 mM EDTA, 50 mM Tris-HCl, 0.5% NP-40, pH 8.0) and incubated with anti-acetyllysine antibody-conjugated beads (PTM0104) at 4°C overnight. After extensive washing, bound peptides were eluted with 0.1% trifluoroacetic acid and desalted using C18 ZipTips prior to LC-MS/MS analysis.

### Molecular modeling

Protein structure prediction was performed using SWISS-MODEL, an automated web-based homology modeling system. The target protein sequence was obtained from the UniProt database, followed by template identification from experimentally determined protein structures. Three-dimensional models were generated through sequence alignment and evaluated based on template identity, amino acid number, and QMEAN score (Patel et al., 2021). The functional domain containing the K6 site was annotated using PyMOL.

## Result

### Determination of succinic acid’s MIC and MBIC against *P.mirabilis*

**Figure 3:**
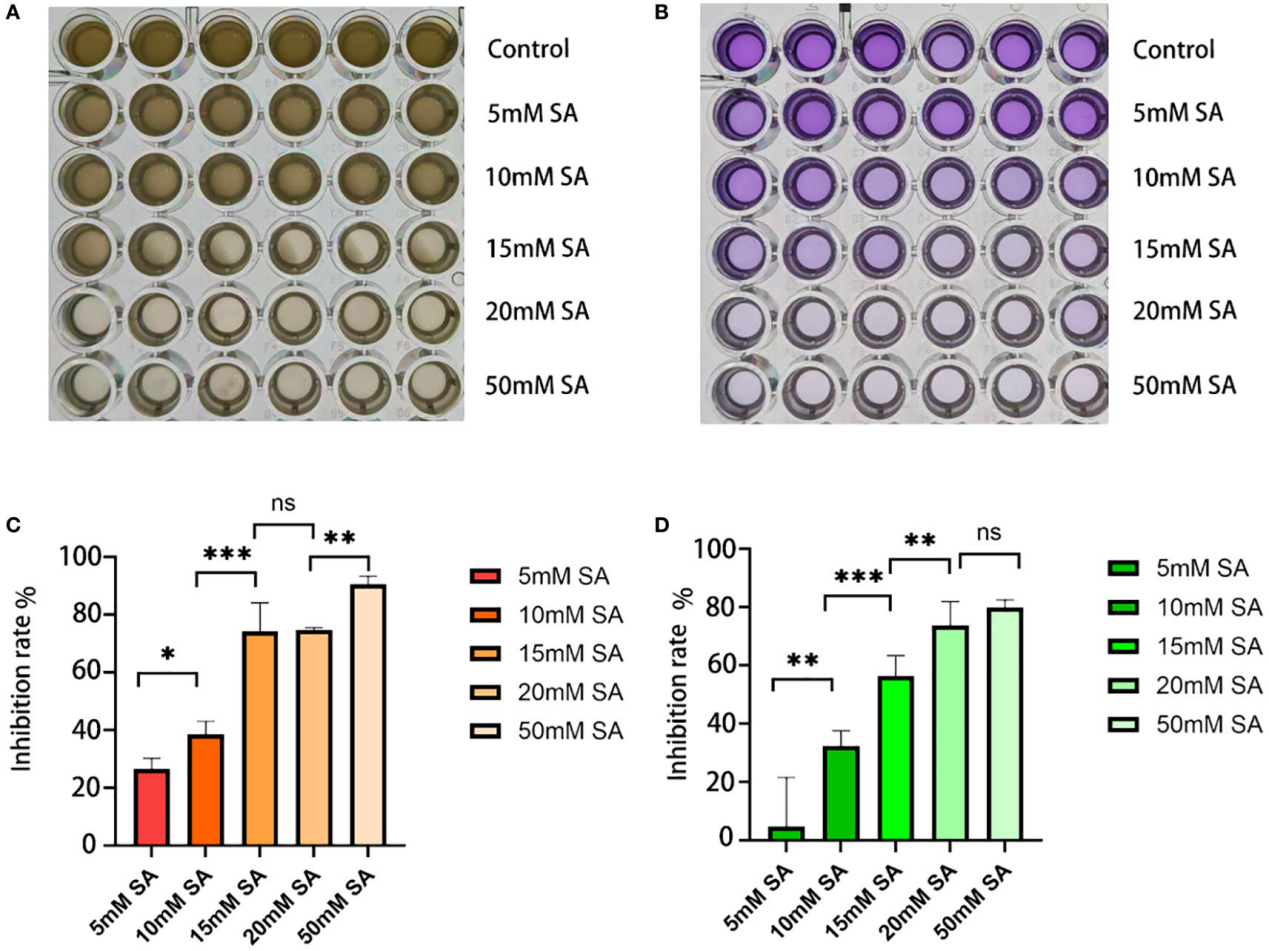
Determination of minimum inhibitory concentration and minimum biofilm inhibitory concentration. **(A, B)** Growth status of *P.mirabilis* in 96-well plates after succinic acid treatment, showing (from top to bottom): control group, 5 mM, 10 mM, 15 mM, 20 mM, and 50 mM (A for MIC, B for MBIC). **(C)** MIC histogram. **(D)** MBIC histogram. The independent-samples t-test was used to compare the differences between each pair of groups for continuous data. * indicates P < 0.05, ** indicates P < 0.01, *** indicates P < 0.001.

The inhibitory effects of succinic acid on *P.mirabilis* are shown in **Figures 3A and B**. In the MIC assay (**Figure 3C**), bacterial growth inhibition significantly increased with rising succinic acid concentrations. The most pronounced inhibitory effect (P < 0.001) was observed at 15 mM, which represented the lowest concentration achieving ≥70% growth inhibition compared to the untreated control, thus establishing 15 mM as the MIC against *P.mirabilis*.

For biofilm formation (**Figure 3D**), succinic acid exhibited concentration-dependent inhibition, with 15 mM demonstrating the most significant suppression (P < 0.001). This concentration achieved >50% biofilm inhibition, thereby being determined as the MBIC against *P.mirabilis*.

### Metabolomics analysis

**Figure 4:**
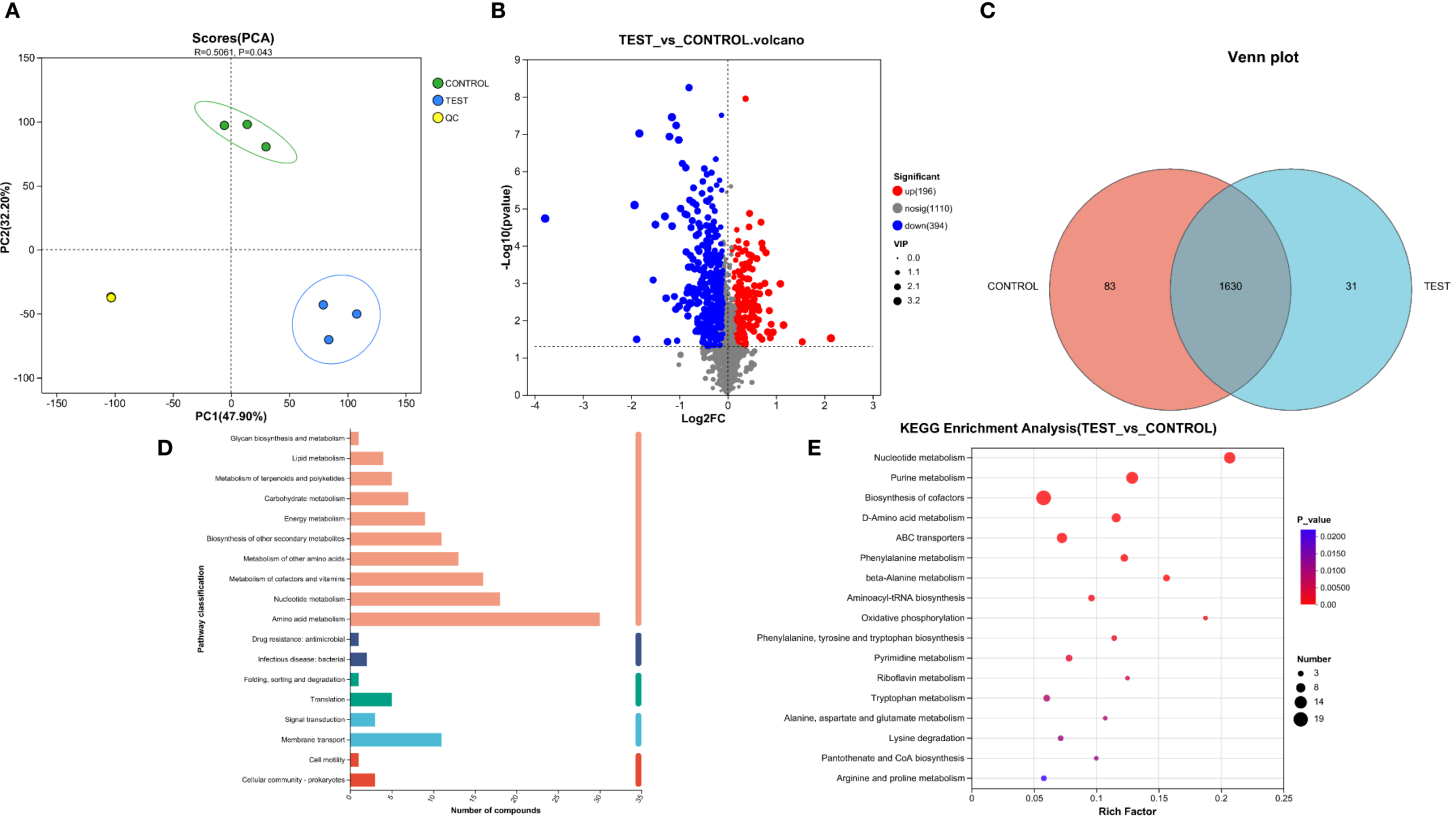
Metabolomic analysis of succinic acid’s inhibition on *P.mirabilis*. **(A)** PCA analysis demonstrated good intra-group reproducibility and significant inter-group separation between the experimental group (MIC concentration of succinic acid treatment) and control group (untreated). **(B)** Volcano plot revealed 196 upregulated and 394 downregulated metabolites in the experimental group compared to controls. **(C)** Venn diagram showed 83 unique metabolites in controls, 31 in treated group, and 1630 shared metabolites. **(D)** Distribution of compounds across different metabolic pathways and biological processes. **(E)** KEGG pathway enrichment analysis.

We performed metabolomic analysis on *P.mirabilis* treated with succinic acid. **Figure 4D** illustrates the distribution of compounds across different metabolic pathways and biological processes, primarily encompassing metabolism, cellular processes, environmental information processing, genetic information processing, and human diseases. Among these, pathways related to amino acid metabolism, nucleotide metabolism, and membrane transport contained a higher number of compounds, whereas processes such as cellular motility and lipid metabolism exhibited fewer. **Figure 4E** presents the KEGG pathway enrichment analysis of the metabolomics data. The differentially abundant metabolites were predominantly enriched in nucleotide metabolism, purine metabolism, and arginine biosynthesis, which are primarily associated with bacterial stress adaptation and metabolic regulatory mechanisms.

Among the various metabolic pathways, those related to amino acid metabolism were particularly critical, as their alterations were directly linked to the regulatory effects of D-amino acids (e.g., L-methionine, L-lysine, L-aspartic acid, linatine) and tryptophan on bacterial physiology. D-amino acids significantly inhibit bacterial growth and disrupt biofilm stability through multiple molecular mechanisms. First, at the protein synthesis level, D-amino acids induce tRNA misacylation and feedback inhibition of key metabolic pathways, leading to ribosomal dysfunction and translational errors. Second, D-amino acids directly interfere with peptidoglycan biosynthesis and are aberrantly incorporated into peptidoglycan side chains, resulting in defective cell wall cross-linking and reduced mechanical strength (Kuru et al., 2012). Collectively, these effects cause bacterial growth arrest and eventual lysis. Regarding biofilm regulation, D-amino acids function via a dual mechanism: at subinhibitory concentrations, they disrupt quorum sensing systems and downregulate the expression of genes encoding biofilm matrix components, such as extracellular polysaccharides and pilin proteins (Leiman et al., 2013); at higher concentrations, D-amino acids are specifically incorporated into mature biofilm structures, displacing D-alanine residues in peptidoglycan peptide bridges, thereby disrupting the anchoring connections between amyloid fibers and cell wall lipoproteins and leading to biofilm structural collapse (Kolodkin-Gal et al., 2010).

The tryptophan metabolic pathway plays a pivotal role in bacterial physiological regulation, with its metabolic network modulating genetic stability, virulence expression, and environmental adaptability through multi-layered molecular mechanisms. Metabolomic analysis revealed significant decreases in key tryptophan pathway intermediates, including indole pyruvate, indole acetate, and tryptamine (**Supplementary Table 1**). Following enzymatic degradation by tryptophanase, tryptophan not only serves as a carbon/nitrogen source for bacteria but also generates indole pyruvate—a critical nodal metabolite that participates in dynamic energy metabolism balance via transamination to produce indole acetate. The depletion of indole pyruvate directly impairs branched-chain amino acid biosynthesis, leading to dysregulated expression of drug resistance-associated proteins (Anyanful et al., 2005), while reduced indole acetate levels compromise its function in stimulating extracellular polysaccharide synthesis, thereby weakening biofilm matrix integrity (Bianco et al., 2006). This metabolic disturbance further extends to quorum sensing systems, where diminished tryptamine—a decarboxylation product of tryptophan—may disrupt neurotransmitter-mimicking mechanisms in bacterial communication (Lee et al., 2007).

The biosynthesis pathways of phenylalanine, tyrosine, and tryptophan occupy central positions in bacterial metabolic networks. By regulating the synthesis of these amino acids, bacteria finely tune intra- and extracellular metabolic homeostasis, influencing essential molecular transport processes, interpopulation signaling, and biofilm formation. Relevant studies demonstrate that these pathways are closely associated with bacterial EPS secretion, which directly governs collective behaviors, antibiotic resistance, and pathogenicity (Guillín et al., 2025). Additionally, these amino acids participate in protein synthesis and energy metabolism. Inhibition or marked reduction of phenylalanine biosynthesis impairs bacterial acquisition and utilization of other essential amino acids, ultimately suppressing growth and proliferative capacity (Zhu et al., 2024).

### Transcriptomics analysis

**Figure 5:**
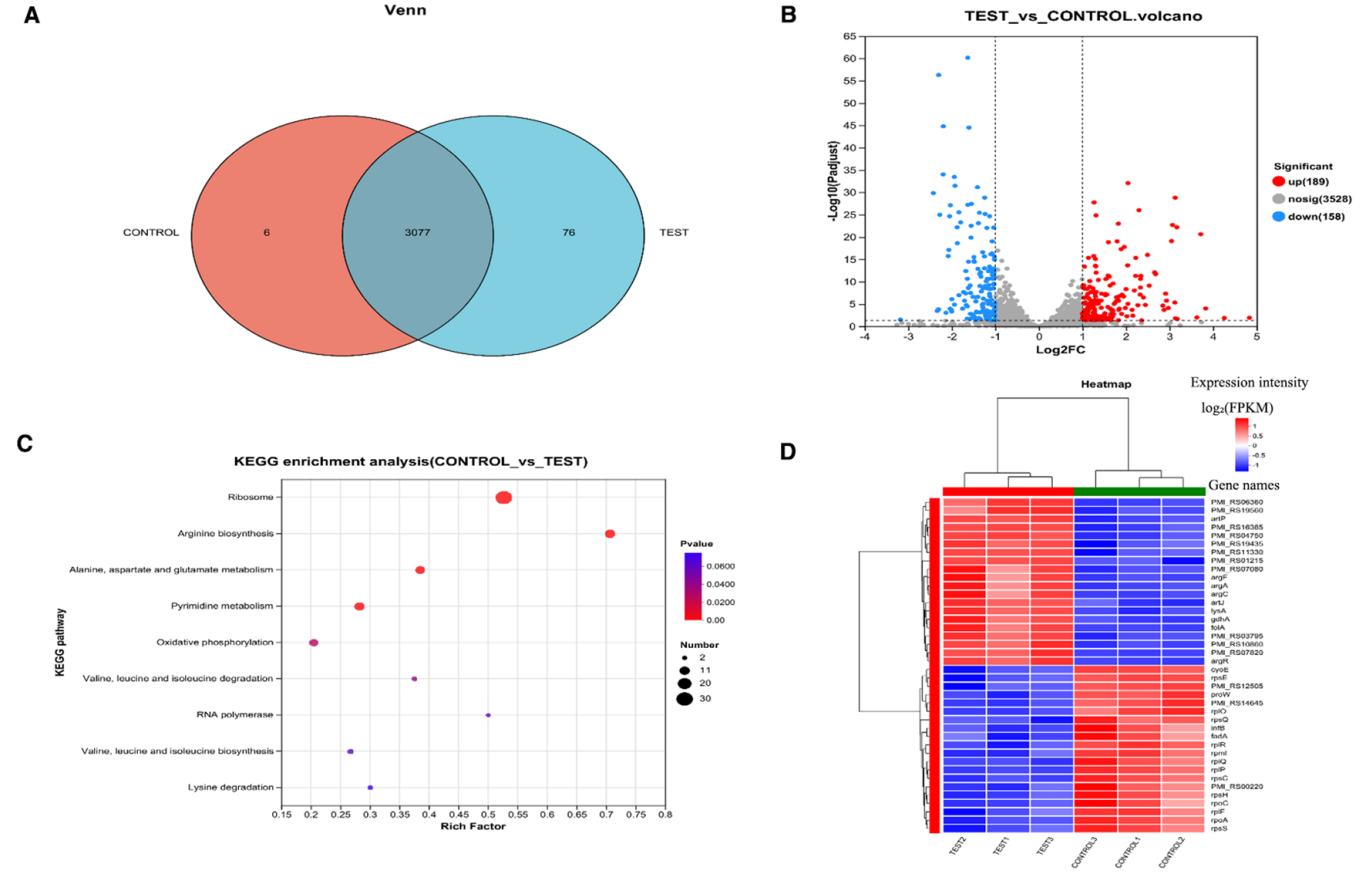
Proteomic analysis of *P. mirabilis* under succinic acid treatment revealed significant alterations in protein expression patterns. **(A)** PCA analysis and **(B)** Pearson correlation coefficients demonstrated high reproducibility among biological replicates. **(C)** Differential protein expression analysis identified 182 significantly upregulated and 252 downregulated proteins, **(D)** with biofilm formation-related pathways being particularly affected as shown in the volcano plot and **(E)** KEGG enrichment analysis. T represents the control group, and H represents the treated group.

KEGG enrichment analysis revealed significant alterations in arginine biosynthesis, glutamine family amino acid metabolism, alanine/aspartate metabolism, nucleotide metabolism, ribosome biogenesis, and oxidative phosphorylation pathways (**Figure 5C**). The heatmap displays the expression patterns of the top 30 DEGs between control and treatment groups (**Figure 5D**). Notably, *artP* and *artJ* genes comprising the arginine transport system cluster *artPIQM-artJ* were markedly upregulated in the succinic acid-treated group. *artP* encodes the ATP-binding protein ArtP of the arginine ABC transporter, which is crucial for transmembrane arginine transport. Given arginine’s importance for bacterial acid resistance, *P.mirabilis* may employ ABC transporters to accumulate osmoprotectants, thereby mitigating osmotic stress by balancing transmembrane gradients (Grigg et al., 2010).

Multiple genes associated with arginine biosynthesis (*argA, argB, gdhA, argH, argE, argC, argF*) and stress response (*pmi_rs16385, pmi_rs04750, pmi_rs11330, pmi_rs06360*) were significantly upregulated (**Supplementary Table 2**), indicating activation of metabolic adaptation and stress response mechanisms following succinic acid treatment. Furthermore, *argR* - encoding the arginine repressor ArgR that negatively regulates arginine biosynthetic genes - showed pronounced upregulation. The *argR* regulon includes its own autoregulatory gene, the *carAB* operon, and genes involved in arginine transport and catabolism (Cho et al., 2015).

Genes encoding 30S and 50S ribosomal subunit proteins (*rplR*, *rplP*, *rpsC*, *rpmI*) were significantly enriched among downregulated genes (**Supplementary Table 2**). Notably, *infB*—which encodes translation initiation factor IF-2—was also markedly downregulated. IF-2 facilitates GTP hydrolysis to GDP and inorganic phosphate (Pi), releasing initiation factors to enable 50S-30S ribosomal subunit association into the 70S initiation complex. The suppression of *infB* inhibits ribosomal biogenesis, thereby blocking protein synthesis and exerting bacteriostatic effects.

Major facilitator superfamily (MFS) transporter genes (*pmi_rs14645, pmi_rs07650, pmi_rs04170, pmi_rs11915*) were significantly downregulated in the succinic acid-treated group. As Gram-negative bacterial efflux pumps, MFS transporters mediate the extrusion of diverse substrates (ions, sugars, amino acids, drugs, and toxins), contributing to multidrug resistance (Du et al., 2018). Additionally, MFS transporters participate in biofilm maturation (May et al., 2009), osmostress protection, and bacterial motility. Prior studies confirm that disrupting MFS transporter genes reduces crystalline biofilm formation in *P.mirabilis* and increases antibiotic susceptibility (Holling et al., 2014).

The NADH:ubiquinone oxidoreductase (respiratory Complex I)—a core enzyme in bacterial energy metabolism—couples NADH oxidation with ubiquinone reduction to drive proton translocation for ATP synthesis. Succinic acid treatment significantly downregulated *nuoI, nuoG, nuoH*, and *nuoJ* (**Supplementary Table 2**): *nuoG*: Stabilizes Complex I via its iron-sulfur cluster-binding sites; its absence causes NDH-1 assembly defects, preventing integration of peripheral subunits (except *nuoC* and *nuoD*) *(* Pohl et al., 2007); *nuoH* and *nuoJ*: Form transmembrane domains housing the quinone-binding site and proton channels; their dysfunction impairs electron transport chain (ETC) efficiency (Friedrich et al., 2016); *nuoI*: A core subunit mediating electron transfer from the NADH dehydrogenase module to the membrane arm; its loss abolishes complex activity (Hedderich and Forzi, 2005). Furthermore, *cyoE*—encoding heme O synthase—was significantly downregulated. This enzyme catalyzes heme O biosynthesis, a critical component of bacterial terminal oxidases O (Mogi et al., 1994). The coordinated downregulation of these genes disrupts ETC electron transfer, proton pumping, and complex assembly, ultimately blocking ATP synthesis and inhibiting bacterial growth.

### Proteomics analysis

**Figure 6:**
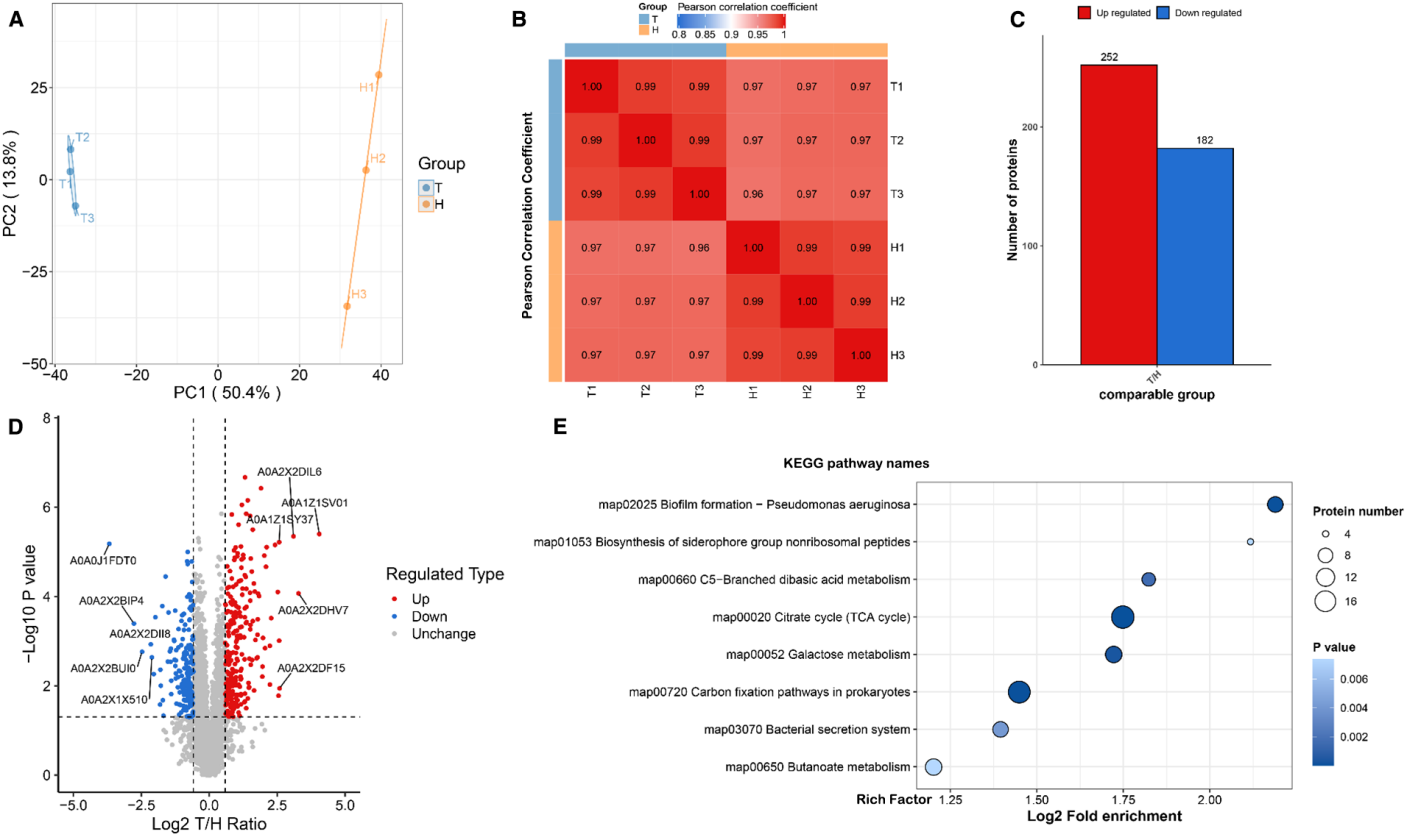
Proteomic analysis of *P.mirabilis* under succinic acid treatment revealed significant alterations in protein expression patterns. **(A)** PCA analysis and **(B)** Pearson correlation coefficients demonstrated high reproducibility among biological replicates. **(C)** Differential protein expression analysis identified 182 significantly upregulated and 252 downregulated proteins, **(D)** with biofilm formation-related pathways being particularly affected as shown in the volcano plot and **(E)** KEGG enrichment analysis.

Key components of the Type VI Secretion System (T6SS), including TssN, TssC, TssA, IdrA, TssJ, and TssH, were significantly downregulated (**Supplementary Table 3**, **Figure 6**). The T6SS plays a crucial role in the pathogenesis of *P.mirabilis* by mediating self-recognition and territorial behaviors through interbacterial competition, as well as exerting an indirect influence on swarming motility via strain competition (Saak et al., 2017). Although its direct role in biofilm formation and stress response in *P.mirabilis* has not been fully elucidated, studies in other bacterial species suggest that T6SS may modulate these processes (Xiong et al., 2024). Specifically, TssA is essential for bacterial fitness during pyelonephritis, with *tssA* mutants showing impaired colonization in murine bladder infection models (Debnath et al., 2018). TssC and TssB form heterodimers that assemble into the contractile sheath required for T6SS function, while TssJ (ClpV) mediates ATP-dependent sheath disassembly and its deletion affects motility, biofilm formation, and EPS production (Li et al., 2024). TssN connects the membrane complex to the baseplate through interaction with TssK to facilitate effector translocation (Bongiovanni et al., 2024), and TssH is required for effector secretion and virulence, with mutants exhibiting defects in T6SS assembly and regulation (Asolkar and Ramesh, 2020).

Proteomic analysis revealed that *P.mirabilis* requires three gene clusters (ids, tss, and idr) for self-recognition, with Ids and Idr proteins representing two distinct mechanisms. Ids proteins encode strain-specific identity determinants, while Idr proteins mediate inter-strain competition during population migration (Saak et al., 2017). T6SS is essential for secreting these proteins, enabling self-recognition and territorial behaviors. Through direct cell contact, T6SS delivers effectors into target cell periplasm via its contractile puncturing apparatus, inducing cell death (Hood et al., 2010). This contact-dependent delivery mechanism underlies its bactericidal function. Importantly, T6SS contributes to catheter-associated urinary tract infection progression (Armbruster et al., 2017), suggesting that succinic acid may inhibit biofilm formation by modulating T6SS-related protein secretion or function.

Additionally, the TCA cycle was significantly enriched in KEGG analysis, with key enzymes downregulated (**Supplementary Table 3**, **Figure 6**). In *Candida albicans*, the TCA cycle is crucial for mature biofilm formation (Sun et al., 2022), influencing matrix synthesis/modification and structural stability. Disrupted TCA cycle enzymes increase dead cells and reduce biofilm biomass, indicating its role in maintaining metabolic homeostasis and biofilm integrity (De Backer et al., 2018). *Bacillus subtilis* studies show TCA intermediate accumulation during early biofilm formation (12–16 hours) enhances metabolic activity, providing material/energy for growth. These intermediates correlate with extracellular matrix (ECM) synthesis, coinciding with upregulated ECM gene expression and biosynthetic precursor levels (Pisithkul et al., 2019), Proteomic analysis further demonstrated significant downregulation of the siderophore biosynthesis system, including key genes *nrpS* (nonribosomal peptide synthase) and *nrpU* (siderophore biosynthesis gene).This specific impairment of siderophore production will critically impair *P.mirabilis* pathogenicity and environmental adaptation (**Supplementary Table 3**). The Nrp system maintains bacterial survival under iron-limited host conditions through competitive iron chelation and transport mediated by siderophores (Armbruster et al., 2018). Dysfunction of this biosynthetic system directly compromises iron acquisition capacity, leading to impaired virulence factor expression and disruption of critical pathogenic processes including swarming motility regulation and flagella synthesis. Mouse infection models confirmed the essential role of the Nrp system in host colonization, with mutants showing significantly reduced adherence in bladder and kidney tissues (Schaffer and Pearson, 2015). Furthermore, dysregulation of iron metabolism-related genes disrupts redox homeostasis, inhibits activation of iron acquisition systems under iron-restricted conditions, and weakens bacterial competition against host iron-binding proteins, ultimately impairing metabolic balance and environmental adaptability (Malik and Hedrich, 2021).

### Exploration of therapeutic targets

Although the direct contribution of T6SS to biofilm formation in *P.mirabilis* is not yet definitive, our data suggest that succinic acid-induced downregulation of T6SS components may indirectly impair biofilm development by disrupting bacterial competition and microcolony architecture. Furthermore, the proposed deacetylation of H-NS and enhanced repression of T6SS genes represent a novel regulatory mechanism that could potentially influence a network of virulence factors, including those involved in biofilm formation.

The histone-like nucleoid structuring protein (H-NS) functions as a global transcriptional regulator that modulates bacterial environmental adaptation through selective silencing of virulence genes. In *Edwardsiella*, H-NS has been shown to repress T6SS gene expression by binding AT-rich promoter regions of T6SS gene clusters and forming transcriptional silencing complexes (Ma et al., 2022). The molecular activity of H-NS is determined by its domain architecture: the N-terminal oligomerization domain mediates self-assembly into functional dimers/tetramers, while the C-terminal DNA-binding domain specifically recognizes target gene promoters (Blot et al., 2006). Notably, the transcriptional repression activity of H-NS is highly dependent on its oligomeric state, as non-oligomerized monomers lack the cooperative binding capacity required for effective RNA polymerase inhibition (Liu et al., 2021). However, whether H-NS regulates T6SS genes through a similar mechanism in *P.mirabilis* remains unreported, presenting a key mechanistic avenue for investigation in this study.

Bacteria dynamically modulate H-NS function via post-translational modifications (PTMs) to adapt to environmental stress (Macek et al., 2019). While existing research on succinic acid’s effects on bacterial phenotypes has primarily focused on succinylation (Wu et al., 2025), studies of H-NS PTMs have largely examined phosphorylation (Liu et al., 2021). Recently, acetylation modifications have emerged as a research focus in metabolite-epigenetic regulation due to their close coupling with central metabolism. Studies reveal that acetylation at K120 within the DNA-binding domain of H-NS abolishes its transcriptional repression activity (Liu et al., 2024). Nevertheless, the functional consequences of acetylation in the N-terminal oligomerization domain and its mechanistic links to metabolic signaling remain unexplored.

This study reveals for the first time that succinic acid specifically modulates the post-translational modification status of *P.mirabilis* H-NS. Acetylomic analysis demonstrated a significant decrease in ϵ-amino acetylation at lysine 6 (K6) of H-NS in succinic acid-treated groups compared to controls (**Supplementary Table 4**). Structural modeling (SWISS-MODEL, WP_368934229.1) and PyMOL annotation localized the K6 site within the N-terminal oligomerization domain of H-NS (**Supplementary Figure 1A**). Functional enrichment analysis further indicated that differentially acetylated proteins were predominantly associated with protein complex oligomerization and tetramerization processes, suggesting succinic acid may influence H-NS function by coordinately regulating multiprotein complex assembly (**Supplementary Figure 1B**).

**Figure 7:**
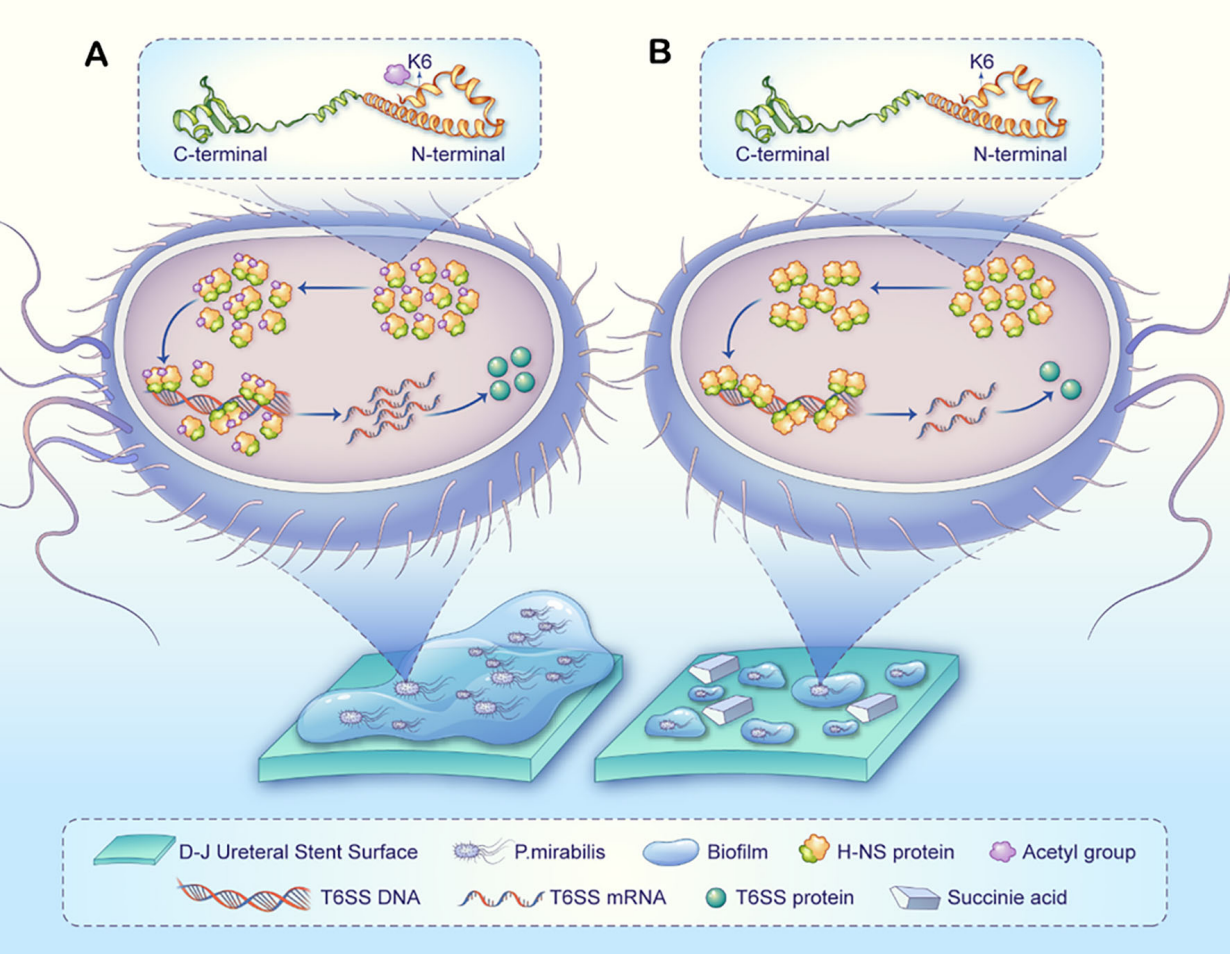
A proposed model illustrating how succinic acid-induced H-NS deacetylation may regulate T6SS to influence *P.mirabilis* biofilm formation on D-J stents. **(A)** Normal biofilm formation process in *P.mirabilis*: The H-NS protein at the K6 site exhibits normal acetylation and oligomerization levels, suppressing the transcription and expression of T6SS-related genes, ultimately leading to biofilm formation. **(B)** In *P.mirabilis* treated with succinate (compared to untreated), the H-NS protein at the K6 site shows reduced acetylation, leading to higher oligomerization, enhanced dimer/tetramer formation, and stronger transcriptional repression of T6SS-related genes. Consequently, fewer T6SS-related proteins are produced, ultimately reducing biofilm formation. It should be noted that biofilm formation is a complex, multifactorial process, and succinic acid may additionally influence this process through broader mechanisms.

Based on these findings, we propose a cascade regulation hypothesis: Succinic acid specifically reduces K6 acetylation of H-NS, inducing conformational rearrangement in its N-terminal oligomerization domain. This promotes the formation of highly stable H-NS tetramers with enhanced binding affinity for AT-rich promoter regions of T6SS gene clusters, thereby strengthening transcriptional repression of T6SS-related genes (**Figure 7**).

## Discussion

This study employed focused metabolomics, transcriptomics, proteomics, and acetylomics to elucidate the inhibitory effects of succinate on *P.mirabilis*—a key pathogen in CAUTIs—and to identify core regulatory nodes under succinate stress. Through targeted multi-omics analyses, we reliably identified key drivers and demonstrated that succinate disrupts bacterial growth and biofilm formation by modulating central metabolic pathways, inhibiting transcription, and altering post-translational modifications. These findings provide novel insights into the molecular interplay between metabolic perturbation and bacterial pathogenicity regulation, offering potential strategies against drug-resistant pathogens.

Succinate exhibits multimodal antibacterial advantages, acting across metabolic, transcriptional, proteomic, and epigenetic regulatory layers to form a synergistic inhibitory network that significantly reduces resistance risks. Our metabolomics analysis revealed significant depletion of tryptophan pathway intermediates alongside global amino acid metabolic dysregulation. This aligns with transcriptomic data showing downregulation of ribosomal genes (*rplR*, *rpsC*) and ATP synthase subunits (*atpD/G/H*). As an essential amino acid, disrupted tryptophan metabolism may impair protein synthesis by limiting translation substrates, disrupting quorum sensing, or interfering with cofactor synthesis, collectively leading to translational arrest, energy imbalance, and compromised biofilm matrix stability.

Notably, succinate suppresses the tricarboxylic acid cycle and oxidative phosphorylation, markedly reducing metabolic flux. Proteomics data further indicate significant downregulation of virulence-associated T6SS core components and iron acquisition system proteins (*NrpS*, *NrpU*), suggesting that metabolic disruption weakens virulence factor production and iron homeostasis, jointly inhibiting biofilm formation. Acetylomics data reveal that succinate induces H-NS deacetylation at K6, potentially enhancing its oligomerization and binding affinity to AT-rich promoter regions of T6SS gene clusters, thereby reinforcing transcriptional repression. This finding links succinate-induced metabolic stress to H-NS-mediated epigenetic regulation.

Traditional antibiotics exert their effects by targeting a single mechanism, such as inhibiting cell wall synthesis (β-lactams), blocking DNA replication (quinolones), or interfering with translation (macrolides). However, this single-target strategy is prone to inducing resistance. In recent years, significant progress has been made in understanding bacterial antibiotic resistance mechanisms, which include the following specific pathways: 1. Enzymatic modification of antibiotics with chemical groups to prevent their binding to bacterial targets (Wright, 2005); 2. Downregulation of porins, reducing antibiotic uptake into cells (Fernández and Hancock, 2012); 3) Bacterial production of a new target protein that binds to the antibiotic (Wilson et al., 2020); 4. Increased expression of efflux pumps to expel antibiotics from bacterial cells (Webber and Piddock, 2003); 5. Protection of ribosomes through mechanisms such as antibiotic removal, enabling ribosomes to evade drug inhibition (Thaker et al., 2010). Our transcriptomics results demonstrate that succinate simultaneously downregulates the expression of ribosomal proteins and MFS proteins, effectively counteracting these bacterial antibiotic resistance mechanisms.

Compared to traditional antibiotics, succinate’s multimodal mechanism offers distinct advantages. Unlike single-target drugs that readily induce resistance, succinate disrupts *P.mirabilis* through a synergistic multi-mechanism network. Fluoxetine and thioridazine, as efflux pump inhibitors, suppress *P.mirabilis* efflux activity, significantly reducing the rate of crystalline biofilm formation on catheters and markedly impairing *P.mirabilis* swimming and swarming motility (Nzakizwanayo et al., 2017). Current research indicates that adjuvants can effectively block antibiotic enzymatic degradation or promote intracellular accumulation, partially overcoming bacterial resistance (Williford et al., 2023). This multi-target intervention logic targeting bacterial core survival mechanisms is highly consistent with current cutting-edge antimicrobial strategies, providing important insights for addressing the challenge of antimicrobial resistance.

Specifically, interventions targeting bacteria’s own essential physiological processes are more effective in preventing the emergence and development of antimicrobial resistance. Antibiotics conjugated with siderophores can exploit bacteria’s inherent requirement for iron, enabling active uptake via specific outer membrane receptors. This not only bypasses β-lactamase degradation and outer membrane barriers but also ensures precise delivery of the antibiotics to their sites of action (Tillotson, 2016). Bacterial metallophore systems like siderophores and zincophores are critical targets for antimicrobial intervention. Interventions targeting these systems, whether by inhibiting their biosynthesis, blocking their secretion or disrupting their metal-binding capability, can perturb bacterial metal ion homeostasis. As a core foundation for bacterial survival and virulence, this disrupted homeostasis ultimately suppresses the colonization of drug-resistant strains (Ezzeddine and Ghssein, 2023). This aligns well with the mode of action of succinate: both approaches act by targeting bacterial “essential physiological processes” rather than “single protein targets,” essentially representing a systemic intervention on bacterial survival mechanisms.

Based on the decoded multimodal mechanism of succinate in this study, future exploration of combined strategies—pairing succinate with adjuvants or antibiotics—could not only reduce antibiotic efflux and enhance drug retention but also restore bacterial antibiotic susceptibility, achieving a triple antimicrobial effect: “enzyme inhibition–efflux blockade–sensitivity restoration.” This multi-target combinatorial approach may overcome the resistance limitations of traditional therapies, offering a more sustainable solution for drug-resistant infections. Amid the increasing emergence of super-resistant pathogens such as carbapenem-resistant Enterobacteriaceae and pan-drug-resistant strains, the application of succinic acid as an antimicrobial agent may represent a promising therapeutic strategy.

This study offers initial insights into the antibacterial mechanism of succinic acid, yet several limitations remain. The clinical tolerability and potential host cytotoxicity of the effective concentration have not been assessed, and biofilm analyses lack structural and compositional characterization. Moreover, the mono-omics approach limits comprehensive understanding of multi-level biological interactions. The proposed regulation of H-NS K6 deacetylation by succinic acid and its causal effect on T6SS require further validation via mutagenesis, ChIP-seq, and complementation assays. The potential influence of succinic acid on other post-translational modifications and their crosstalk with acetylation also remains unexplored. Finally, direct comparisons with standard antibiotics or other organic acids are needed to better contextualize its efficacy. These limitations highlight future directions, including systematic safety evaluation, integration of multi-omics and imaging technologies, mechanistic validation, and comparative efficacy studies to fully support its translational potential.

## Conclusion

This study identifies succinate as a multifaceted inhibitor of *P.mirabilis* pathogenicity, acting through metabolic, transcriptional, and epigenetic pathways. By circumventing the resistance pitfalls of conventional single-target antibiotics, our findings provide a novel perspective for anti-biofilm therapeutic strategies. Future studies should refine succinate-based therapies or inspire the development of synthetic analogs targeting biofilm-associated infections.

## Data Availability

The data presented in the study are as follows: the metabolomics data are deposited in the MetaboLights repository, accession number MTBLS12952; the transcriptomic data are deposited in the NCBI database, accession number PRJNA1320930; the proteomics and acetylomics data are deposited in the ProteomeXchange Consortium via the iProX partner repository, accession number PXD068166.
