# Supplementary material for: Decoding the regulatory networks of Proteus mirabilis under succinic acid stress: a multi-omics approach
Source: Front Cell Infect Microbiol. 2025 Oct 3;15:1650340. doi: 10.3389/fcimb.2025.1650340 (PMC12531150; doi:10.3389/fcimb.2025.1650340)
Supplement: Supplementary file 1 [file DataSheet1.docx]

Supplementary Table.1 Some significantly differentially expressed metabolites in metabolomics.

| Metabolite | FC(TEST/CONTROL) | P_value | KEGG Pathway ID |
| --- | --- | --- | --- |
| 2-Keto-4-methylthiobutyric acid | 0.7515 | 4.29E-05 | map01100;map01210;map01110;map00470;map00270 |
| 3-(3-indolyl)-2-oxopropanoic acid | 0.8543 | 0.000494 | map01100;map00380 |
| 3',5'-Cyclic GMP | 1.1644 | 7.34E-05 | map01100;map00230 |
| 4-hydroxyphenylpyruvic acid | 0.8466 | 0.000455 | map01100;map01110;map01240;map01210;map00130;map00261;map00998;map01230;map00350;map01055;map00400;map00401 |
| 5-hydroxyindole-3-acetic acid | 0.7544 | 0.000154 | map01100;map00380 |
| Acrasin | 1.1811 | 0.03055 | map01100;map00230;map02026;map02025;map05133;map05111;map05110 |
| Adenosine | 1.1644 | 0.001655 | map01100;map02010;map01232;map00230 |
| Adenosine 3'-monophosphate | 1.1583 | 0.000478 | map01100;map00230 |
| Adenosine 5'-diphosphate | 1.2166 | 0.001336 | map01100;map01110;map00230;map01240;map01232;map00195;map00190 |
| Adenosine monophosphate | 1.1389 | 0.02987 | map01100;map01110;map01232;map00230;map01240 |
| beta-Alanine | 1.1669 | 0.001201 | map01100;map01110;map00770;map01240;map00240;map00640;map00410 |
| Cytosine | 1.2003 | 0.001262 | map00240;map01100;map01232 |
| Deoxyguanylic Acid | 1.1899 | 0.01038 | map01100;map01232;map00230 |
| Desthiobiotin | 0.888 | 1.74E-06 | map01100;map00780;map01240 |
| Flavin mononucleotide | 0.879 | 0.01587 | map01100;map01110;map01240;map00998;map01057;map00190;map00740 |
| Gamma-Aminobutyric Acid | 1.0991 | 0.00052 | map01100;map01120;map00650;map02024;map00760;map00330;map00250;map00410 |
| Guanosine | 1.0833 | 0.01183 | map01100;map02010;map01232;map00230 |
| Indole-3-acetic acid | 0.8739 | 0.000498 | map01100;map00380 |
| Inosine | 1.097 | 0.01003 | map01100;map02010;map01232;map00230 |
| L-Aspartic Acid | 1.1689 | 0.004405 | map00997;map01240;map00270;map01200;map00998;map00999;map00250;map00410;map02030;map00770;map02010;map00710;map00470;map01100;map00220;map00300;map01120;map01210;map00340;map01230;map00260;map00261;map01110;map00760;map00460;map02020;map00970 |
| L-Phenylalanine | 0.9212 | 0.01356 | map01100;map00996;map00997;map01210;map02010;map00999;map01230;map00460;map01110;map00470;map00970;map00400;map00360 |
| L-Tryptophan | 0.8977 | 0.02472 | map01100;map00996;map00997;map00260;map01240;map01210;map00999;map01230;map01110;map00970;map00400;map00380;map00404 |
| N-acetylornithine | 1.2487 | 0.000431 | map01100;map01210;map00220;map01110;map01230 |
| Pantothenic acid | 0.7309 | 0.000103 | map01100;map01110;map00770;map00410;map01240 |
| Phenylpyruvic acid | 0.7702 | 0.000288 | map01100;map01110;map01210;map00470;map01230;map00360;map00400 |
| Protoporphyrin IX | 0.8357 | 0.004512 | map01100;map01110;map00860;map01240 |
| Riboflavin | 0.8921 | 0.003305 | map01100;map01240;map01110;map02010;map00740 |
| Thymidine 5'-monophosphate | 1.2033 | 0.02321 | map00240;map01100;map01232 |
| Tryptamine | 0.913 | 3.13E-08 | map01100;map01110;map00380 |
| Uridine | 1.1689 | 0.001836 | map00240;map01100;map02010;map01232 |
| Uridine Monophosphate | 1.261 | 0.000431 | map00240;map01100;map01232;map01240 |
| Xanthosine 5'-monophosphate | 4.3905 | 0.03019 | map01100;map01110;map01232;map00230 |

Supplementary Table.2 Some significantly differentially expressed genes in transcriptomics.

| Gene name | FC(TEST/CONTROL) | P_value | KEGG Pathway ID |
| --- | --- | --- | --- |
| carA | 6.413 | 2.08E-12 | map00240;map00250 |
| carB | 3.564 | 1.83E-06 | map00240;map00250 |
| PMI_RS00220 | 0.377 | 2.33E-25 | map00240;map00230 |
| PMI_RS00230 | 0.494 | 1.11E-15 | map00240;map01232;map00230;map00760 |
| cyoE | 4.92 | 4.03E-26 | map00190;map00860;map04714 |
| rpsA | 4.131 | 9.74E-23 | map03010 |
| pyrD | 2.878 | 5.84E-08 | map00240 |
| rpmI | 0.341 | 3.25E-45 | map03010 |
| PMI_RS05895 | 2.277 | 0.029925995 | map00460;map00250 |
| PMI_RS07200 | 2.3 | 5.44E-06 | map00240;map01232;map00983 |
| pyrC | 2.71 | 4.98E-08 | map00240 |
| nrdA | 0.481 | 2.42E-11 | map00240;map01232;map00230 |
| nrdB | 0.389 | 8.30E-24 | map00240;map01232;map00230 |
| nuoJ | 0.498 | 6.22E-06 | map00190 |
| nuoI | 0.396 | 8.08E-13 | map00190 |
| nuoH | 0.457 | 5.13E-08 | map00190 |
| nuoG | 0.489 | 3.00E-10 | map00190 |
| PMI_RS08655 | 2.696 | 7.13E-05 | map00220;map00250;map00290 |
| argA | 0.457 | 6.58E-23 | map00220 |
| rplA | 0.452 | 5.59E-10 | map03010 |
| PMI_RS13935 | 4.004 | 4.88E-09 | map00220;map00300 |
| gdhA | 0.342 | 1.02E-26 | map00220;map00250;map00910 |
| atpH | 0.439 | 1.89E-06 | map00195;map00190 |
| atpG | 8.393 | 2.32E-16 | map00195;map00190 |
| atpD | 0.34 | 9.30E-20 | map00195;map00190 |
| PMI_RS15180 | 0.449 | 4.63E-13 | map00195;map00190 |
| rpmG | 0.46 | 0.029774355 | map03010 |
| argE | 2.245 | 7.38E-06 | map00220 |
| argC | 3.477 | 8.44E-20 | map00220 |
| argB | 5.28 | 3.42E-07 | map00220 |
| PMI_RS16015 | 5.013 | 8.11E-08 | map00220;map00250;map05418 |
| argH | 2.993 | 4.58E-05 | map00220;map00250 |
| rpsJ | 0.496 | 0.000269206 | map03010 |
| rplC | 0.338 | 3.45E-08 | map03010 |
| rplD | 0.335 | 7.41E-10 | map03010 |
| rplW | 0.38 | 1.38E-13 | map03010 |
| rplB | 0.358 | 6.72E-23 | map03010 |
| rpsS | 2.407 | 8.11E-28 | map03010 |
| rplV | 0.236 | 2.55E-19 | map03010 |
| rpsC | 8.99 | 1.66E-45 | map03010 |
| rplP | 0.273 | 5.62E-57 | map03010 |
| rpmC | 0.356 | 1.89E-16 | map03010 |
| rpsQ | 0.323 | 1.11E-25 | map03010 |
| rplN | 0.458 | 0.000517336 | map03010 |
| rplX | 0.459 | 1.89E-07 | map03010 |
| rplE | 13.156 | 3.43E-15 | map03010 |
| rpsN | 0.488 | 3.14E-23 | map03010 |
| rpsH | 0.242 | 3.94E-28 | map03010 |
| rplF | 0.28 | 3.06E-26 | map03010 |
| rplR | 0.425 | 7.64E-61 | map03010 |
| rpsE | 0.288 | 3.36E-32 | map03010 |
| rpmD | 3.7 | 7.94E-18 | map03010 |
| rplO | 0.438 | 3.56E-34 | map03010 |
| rpsK | 0.46 | 4.68E-09 | map03010 |
| rpsD | 0.478 | 2.81E-12 | map03010 |
| rplQ | 2.46 | 6.39E-28 | map03010 |
| rpsR | 0.455 | 3.21E-09 | map03010 |
| pyrI | 3.957 | 7.99E-05 | map00240;map00250 |
| pyrB | 3.13 | 0.001951508 | map00240;map00250 |
| argF | 0.476 | 1.09E-16 | map00220 |
| gltB | 2.439 | 7.68E-06 | map00250;map00910 |
| ureA | 3.283 | 0.001894666 | map00220;map00230;map00791 |
| PMI_RS18330 | 2.199 | 0.028909607 | map00220;map00230;map00791 |
| rpmJ | 0.48 | 9.69E-06 | map03010 |

Supplementary Table.3 Some significantly differentially expressed proteins in proteomics.

| Gene name | FC(TEST/CONTROL) | P value | KEGG Pathway ID |
| --- | --- | --- | --- |
| acnB_1 | 0.525796 | 0.008395 | map00020; map00630; map00640; map00720; map01100; map01110; map01120; map01200; map01210; map01230 |
| acnB_2 | 0.562021 | 3.95E-05 | map00020; map00630; map00640; map00720; map01100; map01110; map01120; map01200; map01210; map01230 |
| acnB_3 | 0.563703 | 1.44E-06 | map00020; map00630; map00640; map00720; map01100; map01110; map01120; map01200; map01210; map01230 |
| agaD | 0.513139 | 0.000245 | map00052; map01100; map02060 |
| agaF_2 | 0.507172 | 0.001832 | map00052; map01100; map02060 |
| agaS_1 | 0.492203 | 0.000915 | map00052; map01100 |
| agaS_2 | 0.542338 | 0.000338 | map00052; map01100 |
| agaV | 0.431885 | 9.04E-05 | map00052; map01100; map02060 |
| agaW_2 | 0.472828 | 0.009367 | map00052; map01100; map02060 |
| agaZ_2 | 0.535927 | 0.000275 | map00052; map01100 |
| budA | 0.485752 | 0.000761 | map00650; map00660; map01110 |
| budB_1 | 0.578512 | 0.029777 | map00290; map00650; map00660; map00770; map01100; map01110; map01210; map01230 |
| budB_2 | 0.492523 | 0.000578 | map00290; map00650; map00660; map00770; map01100; map01110; map01210; map01230 |
| fdhF_2 | 1.68549 | 0.006457 | map00680; map00720; map01100; map01120; map01200 |
| fdhF_4 | 1.643495 | 0.000183 | map00680; map00720; map01100; map01120; map01200 |
| fdhF_5 | 2.02949 | 0.000351 | map00680; map00720; map01100; map01120; map01200 |
| fdhF_6 | 1.588637 | 9.76E-05 | map00680; map00720; map01100; map01120; map01200 |
| fliA_2 | 2.632901 | 0.007666 | map02020; map02025; map02026; map02040; map05111 |
| gatY | 0.488603 | 1.26E-05 | map00052; map01100 |
| gltA | 0.543048 | 6.02E-05 | map00020; map00630; map01100; map01110; map01120; map01200; map01210; map01230 |
| idrA_1 | 0.29021 | 0.000323 | map02025; map03070 |
| metF_1 | 0.578829 | 0.000134 | map00670; map00720; map01100; map01120; map01200 |
| NCTC10975_00585 | 0.472995 | 0.000175 | map03070 |
| NCTC10975_01154 | 0.516166 | 0.004671 | map00052; map00520; map00541; map01100; map01250 |
| NCTC10975_03227 | 0.254426 | 0.00084 | map02025; map03070 |
| nrpS_1 | 0.575764 | 0.000411 | map01053 |
| nrpS_2 | 0.529869 | 0.000395 | map01053 |
| nrpS_4 | 0.55598 | 0.004224 | map01053 |
| nrpU | 0.517666 | 0.000602 | map01053 |
| pckA_2 | 0.363359 | 0.011035 | map00010; map00020; map00620; map00710; map01100; map01110; map01120; map01200 |
| pckA_3 | 0.486716 | 1.07E-05 | map00010; map00020; map00620; map00710; map01100; map01110; map01120; map01200 |
| ppsA | 0.613878 | 0.000612 | map00010; map00620; map00680; map00720; map01100; map01110; map01120; map01200 |
| rfbD | 0.445524 | 0.025901 | map00052; map00520; map00541; map01100; map01250 |
| sdhA_1 | 0.448632 | 0.00028 | map00020; map00190; map00650; map00720; map01100; map01110; map01120; map01200 |
| sdhA_2 | 0.321324 | 5.12E-05 | map00020; map00190; map00650; map00720; map01100; map01110; map01120; map01200 |
| sdhA_3 | 0.43855 | 1.48E-05 | map00020; map00190; map00650; map00720; map01100; map01110; map01120; map01200 |
| sdhB | 0.421881 | 1.16E-05 | map00020; map00190; map00650; map00720; map01100; map01110; map01120; map01200 |
| sdhD | 0.405339 | 0.000184 | map00020; map00190; map00650; map00720; map01100; map01110; map01120; map01200 |
| sucA_1 | 0.434802 | 8.85E-07 | map00020; map01100; map01110; map01120; map01200 |
| sucA_2 | 0.500972 | 0.000163 | map00020; map01100; map01110; map01120; map01200 |
| sucB_1 | 0.604309 | 6.13E-05 | map00020; map00310; map00380; map01100; map01110; map01120; map01200 |
| sucB_3 | 0.643005 | 0.02329 | map00020; map00310; map00380; map01100; map01110; map01120; map01200 |
| sucC_1 | 0.390145 | 1.39E-06 | map00020; map00640; map00660; map00720; map01100; map01110; map01120; map01200 |
| sucC_2 | 0.401003 | 2.15E-07 | map00020; map00640; map00660; map00720; map01100; map01110; map01120; map01200 |
| sucD_1 | 0.385111 | 0.00037 | map00020; map00640; map00660; map00720; map01100; map01110; map01120; map01200 |
| sucD_2 | 0.491919 | 0.000777 | map00020; map00640; map00660; map00720; map01100; map01110; map01120; map01200 |
| tssA | 0.288108 | 8.61E-05 | map02025 |
| tssC | 0.168193 | 6.07E-06 | map02025 |
| tssH | 0.506295 | 0.000286 | map02025 |
| tssI | 0.307977 | 0.000935 | map03070 |
| tssJ | 0.352898 | 1.55E-06 | map02025; map03070 |
| tssN_1 | 0.539858 | 0.00066 | map02025; map03070 |
| tssN_3 | 0.18728 | 6.94E-06 | map02025; map03070 |

Supplementary Table.4 The ε-amino acetylation site of H-NS lysine 6 (K6).

| Amino acid | Position | H/TSB Ratio | Gene name |
| --- | --- | --- | --- |
| K | 6 | 0. 398406374 | hns |


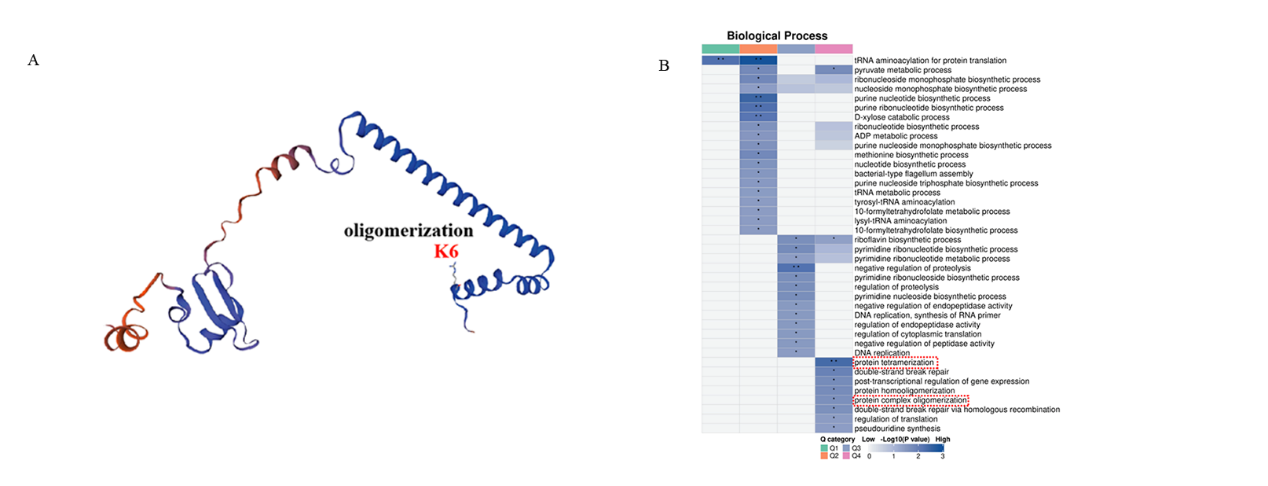


Supplementary Figure.1 (A) Structural domain annotation of the H-NS K6 site via SWISS-MODEL. (B) KEGG functional enrichment analysis of acetylation in *Proteus mirabilis* treated with succinic acid.
